# SlGRAS4 accelerates fruit ripening by regulating ethylene biosynthesis genes and *SlMADS1* in tomato

**DOI:** 10.1038/s41438-020-00431-9

**Published:** 2021-01-01

**Authors:** Yudong Liu, Yuan Shi, Deding Su, Wang Lu, Zhengguo Li

**Affiliations:** 1 Key Laboratory of Plant Hormones and Development Regulation of Chongqing, School of Life Sciences, Chongqing University, 401331 Chongqing, China; 2 Center of Plant Functional Genomics, Institute of Advanced Interdisciplinary Studies, Chongqing University, 401331 Chongqing, China

**Keywords:** Molecular engineering in plants, Transgenic organisms, Transcriptional regulatory elements

## Abstract

GRAS proteins are plant-specific transcription factors that play crucial roles in plant development and stress responses. However, their involvement in the ripening of economically important fruits and their transcriptional regulatory mechanisms remain largely unclear. Here, we demonstrated that *SlGRAS4*, encoding a transcription factor of the GRAS family, was induced by the tomato ripening process and regulated by ethylene. Overexpression of *SlGRAS4* accelerated fruit ripening, increased the total carotenoid content and increased *PSY1* expression in *SlGRAS4*-OE fruit compared to wild-type fruit. The expression levels of key ethylene biosynthesis genes (*SlACS2*, *SlACS4*, *SlACO1*, and *SlACO3*) and crucial ripening regulators (*RIN* and *NOR*) were increased in *SlGRAS4*-OE fruit. The negative regulator of tomato fruit ripening, *SlMADS1*, was repressed in OE fruit. Exogenous ethylene and 1-MCP treatment revealed that more endogenous ethylene was derived in *SlGRAS4*-OE fruit. More obvious phenotypes were observed in OE seedlings after ACC treatment. Yeast one-hybrid and dual-luciferase assays confirmed that SlGRAS4 can directly bind *SlACO1* and *SlACO3* promoters to activate their transcription, and SlGRAS4 can also directly repress *SlMADS1* expression. Our study identified that SlGRAS4 acts as a new regulator of fruit ripening by regulating ethylene biosynthesis genes in a direct manner. This provides new knowledge of GRAS transcription factors involved in regulating fruit ripening.

## Introduction

Fruit ripening can be classified as climacteric or nonclimacteric, depending on the presence or absence, respectively, of massive ethylene production during ripening^1^. Tomato (*Solanum lycopersicum*) is a classic model of climacteric fruit ripening. Many biological changes, including color conversion, softening, and sugar/acidity alteration, occur during the fruit ripening process, and the ethylene burst is closely related to the rise in climacteric respiration. It is important to understand the fruit ripening process that determines the nutritional quality, storage life and wastage of many fresh plant products worldwide.

Ethylene is an important phytohormone for fruit ripening, and ethylene biosynthesis is strictly regulated during fruit ripening. Ethylene biosynthesis is divided into two steps. The first step is the rate-limiting step, in which the conversion of S-adenosyl-l-Met (SAM) to 1-aminocyclopropane-1-carboxylic acid (ACC) is catalyzed by ACC synthase (ACS). Then, the conversion of ACC to ethylene is catalyzed by ACC oxidase (ACO)^2,3^. Fourteen *ACS* genes and 6 *ACO* genes have been identified in tomato, and the expression of *ACS2*, *ACS4*, *ACO1* and *ACO2* was significantly induced by fruit ripening initiation, suggesting that they may act as the main genes for ethylene biosynthesis during tomato fruit ripening^4^. Two ethylene biosynthesis systems, system 1 and system 2, were introduced based on the level of ethylene production during fruit development^5^. Ethylene production exhibited a massive increase associated with fruit ripening in climacteric fruits and was considered system 2. In tomato fruit, ethylene production shifted from system 1 to system 2 at the climacteric stage, with an accumulation of transcripts of *ACS2*, *ACS4*, *ACO1*, and *ACO4*, resulting in positive feedback regulation^6,7^.

In the past few years, many transcription factors involved in fruit ripening have been identified. The natural mutants *rin* (ripening-inhibitor), *nor* (nonripening) and *Cnr* (colorless nonripening) have been widely used for studying the regulatory mechanisms of fruit ripening. RIN is a classic MADS-box transcription factor, and previous studies have suggested that *rin* is a loss-of-function mutant, but a recent study indicated that *rin* is actually a gain-of-function mutant^8^. Regardless of the mechanism, a large number of RIN target genes have been identified, including *SlACS2* and *SlACS4*, as have many other genes that participate in the regulation of ethylene signaling and fruit quality formation during ripening^9–12^. The *nor* mutant exhibited a nonripening phenotype (Patent US 6,762,347 B1)^13^. *NOR* encodes an NAC transcription factor that influences many more genes than *RIN* during fruit ripening^14^. However, unlike the natural mutant, a recent study showed that the ripening progress of knock-out mutants produced by CRISPR/Cas9 was only partly affected^15,16^. Formation of the *Cnr* mutant was caused by the increased cytosine methylation level in the promoter region of the *LeSPL-CNR* gene, and the epigenetic change led to a severe nonripening phenotype^17^. However, a recent study showed that the fruit ripening of *LeSPL-CNR* CRISPR/Cas9 lines was only delayed^18^. Moreover, a large number of other transcription factors influence fruit ripening by regulating ethylene biosynthesis genes, including *SlMADS1*, *FUL1*, *FUL2*, *TAGL1*, *SlMBP8*, and *SlMBP15* belonging to the MADS-box family^19–23^; *SlNAC1* and *SlNAC4* belonging to the NAC family^24,25^; and *SlAP2a* and *SlERF.B3* belonging to the ERF transcription factor family^26,27^.

Several studies have shown that GRAS transcription factors are widely involved in regulating plant development and resisting abiotic stress^28–31^. However, few studies have reported that GRAS participates in regulating fruit ripening, except for a report on *SlGRAS38*-silenced fruit with lower lycopene content and lower ethylene production^32^. The expression of *SlGRAS38*, also named *SlFSR*, increased during fruit ripening, and downregulation of *SlFSR* altered cell wall modification and prolonged fruit shelf life^33^. Our previous study identified that *SlGRAS4* encodes a GRAS transcription factor and plays a positive role in enhancing chilling tolerance in tomato fruit. Fruit with *SlGRAS4* overexpression could ripen normally after chilling treatment, whereas WT fruit could not turn red completely^31^. Here, the expression of *SlGRAS4* was induced by the fruit ripening process, and overexpression of *SlGRAS4* accelerated fruit ripening. We also confirmed that SlGRAS4 can directly bind to and activate the promoters of the ethylene biosynthesis genes *SlACO1* and *SlACO3*, suggesting that SlGRAS4 influences fruit ripening mainly through *SlACO1* and *SlACO3*. Furthermore, SlGRAS4 can also directly repress *SlMADS1* expression, and our study provides new insight into the GRAS transcription factors that are involved in regulating fruit ripening.

## Results

### *SlGRAS4* expression is induced by fruit ripening and exhibits ethylene regulation

**Fig. 1:**
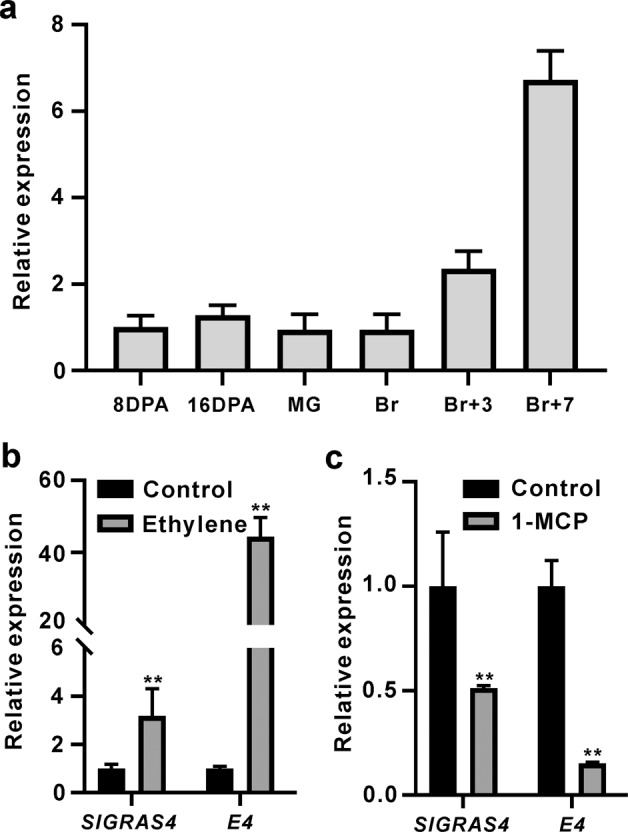
Expression patterns of *SlGRAS4* in wild-type (WT) tomato. **a** Expression patterns of *SlGRAS4* in 8 DPA (days post anthesis) fruit, 16 DPA fruit, mature green fruit (MG), breaker fruit (Br), 3 days post breaker (Br + 3) fruit, and Br + 7 fruit of WT tomato. **b** The relative expression levels of *SlGRAS4* and *E4* in ethylene-treated WT mature green fruit. **c** The relative expression levels of *SlGRAS4* and *E4* in 1-methylcyclopropene (1-MCP)-treated WT breaker fruit. The data represent the mean values of three independent experiments, and error bars show the ±standard error values. In (**b**) and (**c**), ** refers to significant differences between control and treatment with *P* < 0.01 (two-tailed Student’s *t*-test)

To explore the expression pattern of *SlGRAS4* (Soly01g100200) during fruit development and ripening, fruit at different stages were investigated, including 8 DPA (days post anthesis) fruit, 16 DPA fruit, mature green fruit (MG), breaker fruit (Br), 3 days post breaker (Br + 3) fruit, and 7 days post breaker (Br + 7) fruit. The expression level of *SlGRAS4* gradually increased from breaker to red ripe fruit, which accompanied the fruit ripening process (Fig. 1a), suggesting that SlGRAS4 may play a role during fruit ripening in tomato. To investigate whether *SlGRAS4* was under ethylene regulation, wild-type (WT) MG fruit were treated with ethylene, and *SlGRAS4* was significantly induced by ethylene treatment. Simultaneously, *E4*, an ethylene response gene, was used as a control to validate the efficacy of the treatment (Fig. 1b). On the other hand, WT Br was treated with 1-methylcyclopropene (1-MCP), and *SlGRAS4* was significantly repressed after 1-MCP treatment (Fig. 1c), suggesting that *SlGRAS4* was under ethylene regulation.

### Overexpression of *SlGRAS4* accelerates fruit ripening

**Fig. 2:**
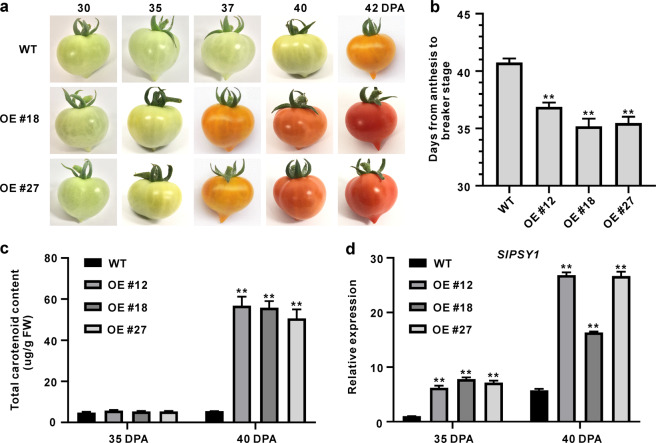
Phenotypic characterization of WT and *SlGRAS4* transgenic fruit. **a** Phenotype of *SlGRAS4-*overexpressing (OE) fruit. The color turning of OE fruit occurred earlier than that of WT fruit. DPA, days post anthesis. **b** Days from anthesis to the breaker stage in WT and *SlGRAS4-*OE fruit. **c** Total carotenoid content in WT and *SlGRAS4*-OE fruit at 35 DPA and 40 DPA. **d** Expression of *SlPSY1* in 35 DPA and 40 DPA fruit of WT and *SlGRAS4-*OE lines. In (**b**) to (**d**), the data represent the mean values of three independent experiments, and error bars show the ±standard error values. ** Refers to significant differences between WT and transgenic lines with *P* < 0.01 (two-tailed Student’s *t*-test)

To investigate the function of *SlGRAS4* in fruit ripening, the days at anthesis and DPA were recorded for WT and *SlGRAS4* overexpression plants (the efficiency of overexpression is shown in Fig. S1). The *SlGRAS4*-OE fruit exhibited earlier ripening than the WT fruit, and the OE fruit showed an orange color at 37 DPA, whereas the WT was still at the mature green stage; when the WT fruit reached the orange stage at 42 DPA, the OE fruit was red ripe (Fig. 2a). The calculated days from anthesis to the breaker stage showed that the ripening period of OE fruit was ~5 days earlier than that of WT fruit (Fig. 2b). The most obvious characteristic of fruit ripening is the color changes that occur with the accumulation of carotenoids. We further measured the total carotenoid content in WT and *SlGRAS4*-OE fruit, and low levels of carotenoids were observed in both WT and OE fruit at 35 DPA, but the carotenoid content in OE fruit was much higher than that in WT at 40 DPA (Fig. 2c). *PSY1* encodes phytoene synthase 1, which plays a critical role in the synthesis of carotenoids. The expression level of *SlPSY1* in OE fruit was much higher than that in WT fruit at both 35 DPA and 40 DPA (Fig. 2d), which is consistent with the high carotenoid level in OE fruit.

### Ethylene biosynthesis and ripening-related genes are induced in *SlGRAS4*-OE fruit

**Fig. 3:**
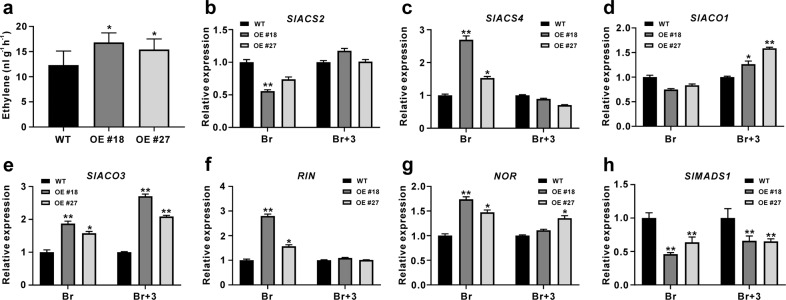
Ethylene production and the expression of ethylene biosynthesis and ripening-related genes in WT and *SlGRAS4* transgenic fruit. **a** Ethylene production in WT and *SlGRAS4*-OE fruit at the breaker stage. The expression levels of *SlACS2* (**b**), *SlACS4* (**c**), *SlACO1* (**d**), *SlACO3* (**e**), *RIN* (**f**), *NOR* (**g**), and *SlMADS1* (**h**) were analyzed by qRT-PCR. Br, breaker stage; Br + 3, 3 days post breaker stage. The data represent the mean values of three independent experiments, and error bars show the ±standard error values. * and ** refer to significant differences between WT and transgenic lines with *P* < 0.05 and *P* < 0.01, respectively (two-tailed Student’s *t*-test)

More ethylene production was found in *SlGRAS4*-OE fruit at the breaker stage than in WT fruit (Fig. 3a). The expression of ethylene biosynthesis genes, ethylene signaling genes and crucial transcription factors in fruit ripening was analyzed in WT and OE fruit (Fig. 3b–h, S2–4). The expression levels of *SlACS4* and *SlACO3* in OE fruit were significantly higher than those in WT at the breaker stage, and the expression levels of *SlACO1* and *SlACO3* in OE fruit were higher than those in WT at the Br + 3 stage (Fig. 3b–e). The expression levels of other ethylene biosynthesis genes, including *SlACS1a*, *SlACS6*, *SlACO2*, *SlACO4*, and *SlSAM1*, showed no obvious changes in OE fruit compared to WT fruit at the breaker stage, except that *SlACS3* was downregulated in OE fruit (Fig. S2). There were no significant differences in most ethylene signaling genes between WT and *SlGRAS4*-OE fruit at the breaker stage, except that *SlEBF3* expression was much higher in OE fruit (Fig. S3). As important regulators of ethylene biosynthesis and fruit ripening, the transcriptional levels of *RIN* and *NOR* were also significantly induced in OE fruit at the breaker stage (Fig. 3f, g). In contrast, the expression of *SlMADS1*, a negative regulator of tomato fruit ripening, was significantly downregulated in OE fruit compared to WT fruit at both the breaker and Br+3 stages (Fig. 3h). In addition, *SlPG2a* and *SlFUL1* were expressed at higher levels in OE fruit at the breaker stage (Fig. S4). These results suggested that SlGRAS4 may accelerate fruit ripening by influencing the expression of genes involved in ethylene biosynthesis and important transcription factors.

**Fig. 4:**
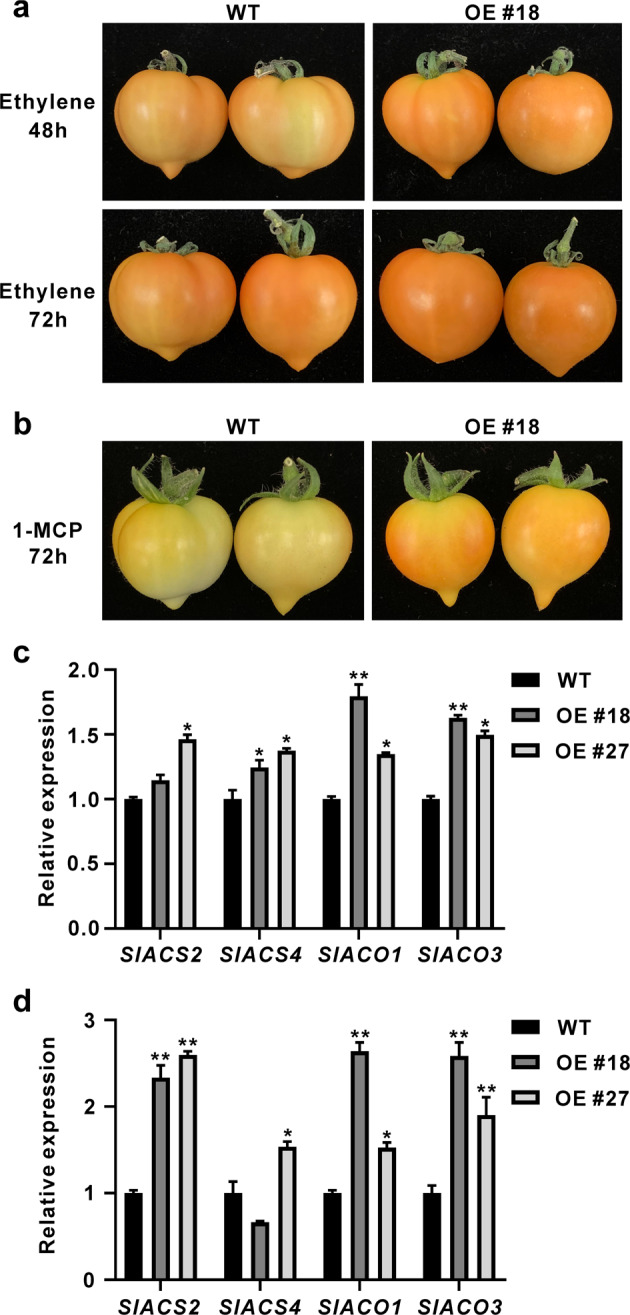
Effect of ethylene and 1-MCP treatment on WT and *SlGRAS4-OE* fruit. **a** Phenotypes of WT and *SlGRAS4* OE #18 fruit after treatment with ethylene for 48 and 72 h, respectively. The fruit were picked at the mature green stage for ethylene treatment. **b** Phenotypes of WT and *SlGRAS4* OE #18 fruit after treatment with 1-MCP for 72 h. The fruit were picked at the breaker stage for 1-MCP treatment. **c** The expression levels of *SlACS2*, *SlACS4*, *SlACO1*, and *SlACO3* in WT and *SlGRAS4-*OE fruit after treatment with ethylene for 72 h. **d** The expression levels of *SlACS2*, *SlACS4*, *SlACO1*, and *SlACO3* in WT and *SlGRAS4-*OE fruit after treatment with 1-MCP for 72 h. In (**c**) and (**d**), the data represent the mean values of three independent experiments, and error bars show the ±standard error values. * and ** refer to significant differences between WT and transgenic lines with *P* < 0.05 and *P* < 0.01, respectively (two-tailed Student’s *t*-test)

### *SlGRAS4*-OE fruit exhibit more obvious phenotypes after exogenous ethylene and 1-MCP treatment

Ethylene is crucial for tomato fruit ripening, and 1-MCP is a potent inhibitor of ethylene perception that can inhibit fruit ripening. WT and *SlGRAS4*-OE fruit at the mature green stage were picked and used for exogenous ethylene treatment. Color turning in OE fruit occurred noticeably earlier than that in WT fruit after treatment for 48 h, and WT and OE fruit both turned orange after treatment for 72 h, but OE fruit looked darker (Fig. 4a). On the other hand, WT and *SlGRAS4*-OE fruit at the breaker stage were picked and used for exogenous 1-MCP treatment. There were obvious differences between WT and OE fruit after 1-MCP treatment. The OE fruit were light orange after treatment for 72 h, whereas the WT fruit were still at the breaker stage and exhibited no obvious color turning (Fig. 4b); in addition, more ethylene was produced in OE fruit (Fig. S5), suggesting that the accelerated color turning in OE fruit after 1-MCP treatment was caused by the higher levels of endogenous ethylene. The expression of four crucial ethylene biosynthesis genes, namely, *SlACS2*, *SlACS4*, *SlACO1*, and *SlACO3*, was higher in OE fruit than in WT fruit after ethylene treatment for 72 h (Fig. 4c). Consistent with the phenotype, the expression levels of *SlACS2*, *SlACS4*, *SlACO1*, and *SlACO3* in OE fruit were all significantly higher than those in WT fruit after 1-MCP treatment (Fig. 4d).

### *SlGRAS4*-OE seedlings exhibit more intense phenotypes after exogenous ACC treatment

**Fig. 5:**
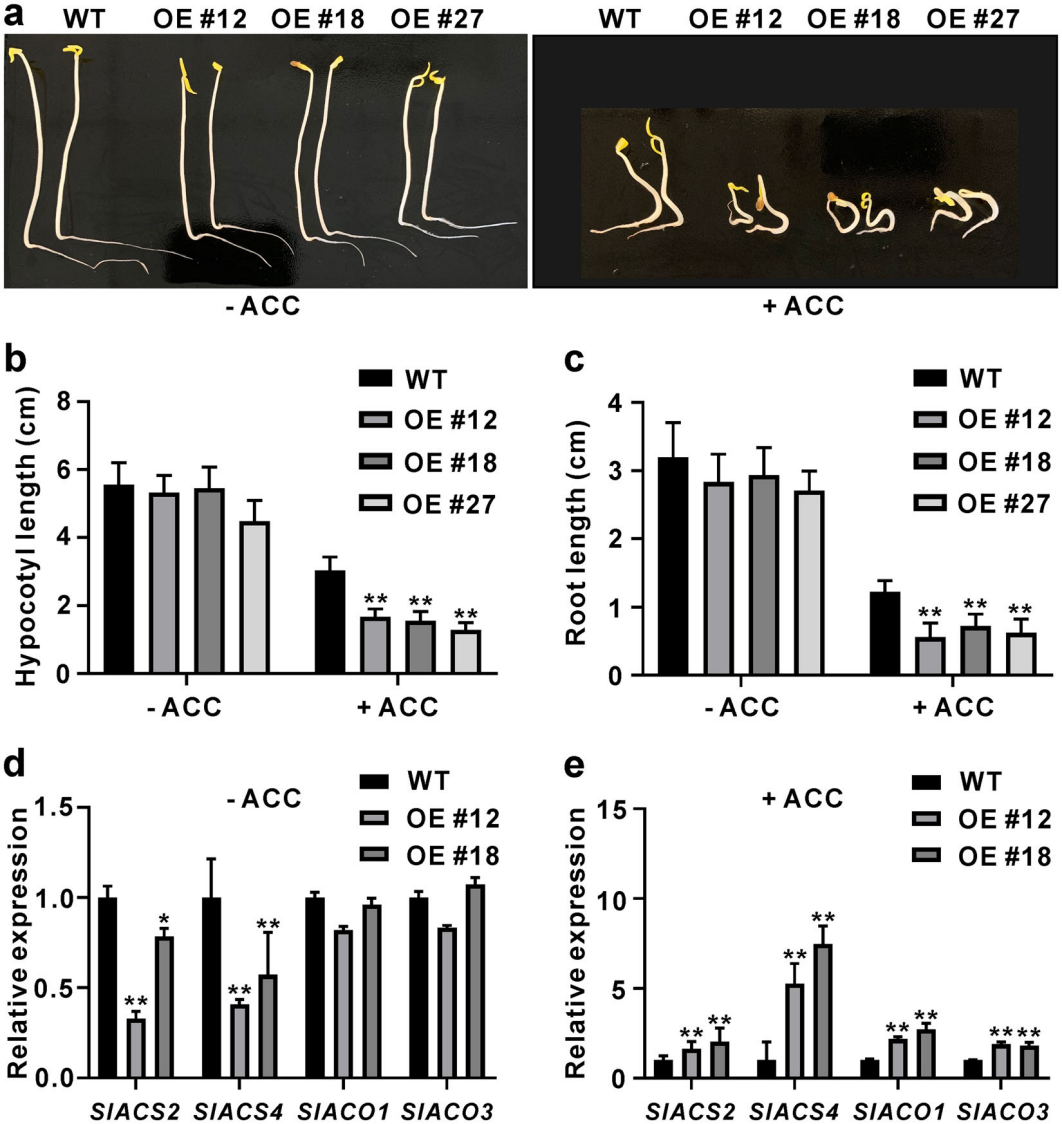
Ethylene triple response of WT and *SlGRAS4*-OE seedlings. **a** Phenotypes of WT and *SlGRAS4*-OE seedlings in the absence or presence of 2.0μM 1-aminocyclopropane-1-carboxylate (ACC). Hypocotyl length (**b**) and root length (**c**) of WT and *SlGRAS4*-OE seedlings in the absence or presence of 2.0 μM ACC. **d** The expression levels of *SlACS2*, *SlACS4*, *SlACO1*, and *SlACO3* in WT and *SlGRAS4-*OE seedlings without ACC treatment. **e** The expression levels of *SlACS2*, *SlACS4*, *SlACO1*, and *SlACO3* in WT and *SlGRAS4-*OE seedlings with ACC treatment. In (**b**) to (**e**), the data represent the mean values of three independent experiments, and error bars show the ±standard error values. * and ** refer to significant differences between WT and transgenic lines with *P* < 0.05 and *P* < 0.01, respectively (two-tailed Student’s *t*-test)

To ascertain whether the increased level of ethylene production persisted in nonfruit tissues in *SlGRAS4*-overexpressing plants, an ethylene triple-response assay was performed. The ethylene precursor 1-aminocyclopropane-1-carboxylate (ACC) was used to treat WT and OE seedlings. In the absence of ACC, there were no significant differences in hypocotyl length and root length between WT and OE seedlings (Fig. 5a–c). Hypocotyl elongation and root elongation were inhibited in both WT and OE seedlings after ACC treatment, but the inhibition in OE seedlings was more severe than that in WT seedlings, and the bending of hypocotyls was more obvious in OE seedlings (Fig. 5a–c), suggesting that more ethylene may be produced in *SlGRAS4*-OE seedlings. Furthermore, the expression of *SlACS2*, *SlACS4*, *SlACO1* and *SlACO3* was detected in WT and *SlGRAS4-*OE seedlings with or without ACC treatment. There were no changes in *SlACO1* and *SlACO3* in OE seedlings in the absence of ACC, whereas *SlACS2* and *SlACS4* were downregulated (Fig. 5d). However, after ACC treatment, all four genes were significantly upregulated in OE seedlings compared to WT seedlings (Fig. 5e). These results provide molecular evidence for the likely increase in ethylene production in *SlGRAS4-*OE seedlings.

### SlGRAS4 directly binds to and activates the *SlACO1* and *SlACO3* promoters

**Fig. 6:**
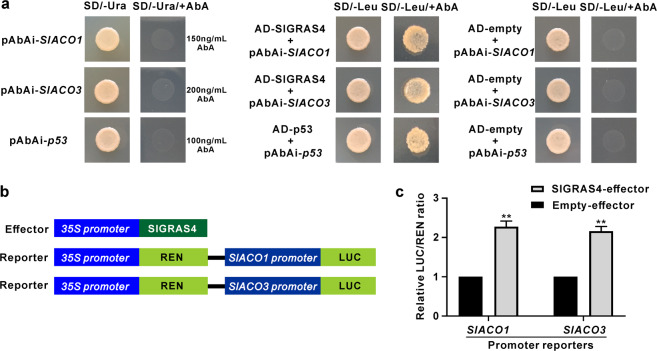
SlGRAS4 directly binds to and activates the *SlACO1* and *SlACO3* promoters. **a** SlGRAS4 binding with *SlACO1* and *SlACO3* promoter fragments assessed by a yeast one-hybrid assay. **b** Effector and reporter constructs used for the dual-luciferase assay. **c** SlGRAS4 activates *SlACO1* and *SlACO3* promoter activity as determined by a dual-luciferase assay. The empty effector was used as a control (set as 1). In (**c**), the data represent the mean values of five independent experiments, and error bars show the ±standard error values. ** Refers to significant differences between the SlGRAS4-effector group and empty group with *P* < 0.01 (two-tailed Student’s *t*-test)

The results described above suggest that SlGRAS4 accelerates fruit ripening by influencing ethylene biosynthesis. The expression of *SlACS2*, *SlACS4*, *SlACO1*, and *SlACO3* was enhanced in OE fruit regardless of whether they were on the vine or treated with ethylene and 1-MCP. On the other hand, several SlGRAS4-binding motifs were identified on the promoters of *SlACS2*, *SlACS4*, *SlACO1*, and *SlACO3* by an *in silico* search (Table S1), suggesting that SlGRAS4 may regulate the expression of these genes in a direct manner. A yeast one-hybrid assay was performed to verify the interaction of SlGRAS4 with these promoter fragments, and the results showed that SlGRAS4 can directly bind to the *SlACO1* and *SlACO3* promoter fragments (Fig. 6a), whereas there was no interaction between SlGRAS4 and the *SlACS2* and *SlACS4* promoters. The dual-luciferase assay further confirmed that SlGRAS4 directly activates the *SlACO1* and *SlACO3* promoters (Fig. 6b, c). These results confirmed that SlGRAS4 influences ethylene biosynthesis by regulating *SlACO1* and *SlACO3* expression in a direct manner.

### SlGRAS4 directly binds to the *SlMADS1* promoter and represses its expression

**Fig. 7:**
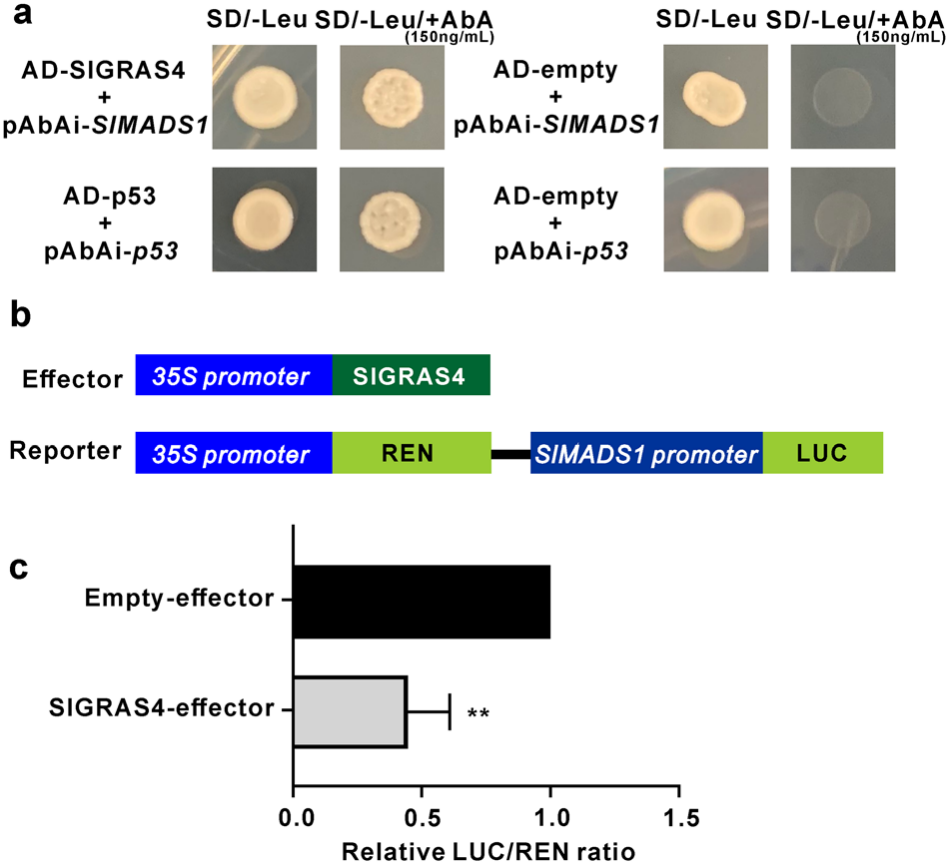
SlGRAS4 directly binds to and represses the *SlMADS1* promoter. **a** SlGRAS4 binding with the *SlMADS1* promoter fragment assessed by a yeast one-hybrid assay. **b** Effector and reporter constructs used for the dual-luciferase assay. **c** SlGRAS4 represses *SlMADS1* promoter activity as determined by a dual-luciferase assay. The empty effector was used as a control (set as 1). In (**c**), the data represent the mean values of five independent experiments, and error bars show the ±standard error values. ** Refers to significant differences between the SlGRAS4-effector group and empty group with *P* < 0.01 (two-tailed Student’s *t*-test)

*SlMADS1* negatively regulates fruit ripening in tomato, and silencing of *SlMADS1* accelerates fruit ripening. *SlACS2*, *SlACO1*, and *SlACO3* were upregulated in *SlMADS1*-silenced fruit^21^. *SlMADS1* was significantly downregulated in *SlGRAS4*-OE fruit during fruit ripening (Fig. 3h). In our previous study, *SlMADS1* was identified as one of the SlGRAS4 target genes through a ChIP-seq approach^31^. Several SlGRAS4-binding motifs on the *SlMADS1* promoter were identified by an *in silico* search (Table S1). A yeast one-hybrid assay showed that SlGRAS4 can directly bind to the *SlMADS1* promoter fragment (Fig. 7a, S6), and a dual-luciferase assay revealed that SlGRAS4 can directly repress the promoter activity of the *SlMADS1* gene (Fig. 7b, c). These results indicated that the accelerated fruit ripening of *SlGRAS4*-OE fruit is also caused by the repression of *SlMADS1* in a direct manner.

## Discussion

Previous studies have revealed that tomato GRAS transcription factors are involved in regulating plant growth and development and participate in regulating abiotic stress resistance^29–31,34–36^. Silencing of *SlGRAS38* significantly lowered the lycopene content and ethylene production in tomato fruit, and several ripening-related genes were influenced by SlGRAS38^32^. In the *SlFSR*-RNAi fruit, the expression of several cell wall modification-related genes was decreased, and the related enzyme activities were decreased, which prolonged the fruit shelf life, and overexpression of *SlFSR* in *rin* resulted in upregulation of multiple cell wall modification-related genes and shortened fruit shelf life^33^. Moreover, there is no other study on GRAS regulating fruit ripening. Here, we identified that *SlGRAS4* was induced by the fruit ripening process (Fig. 1), and overexpression of *SlGRAS4* accelerated fruit ripening (Fig. 2a). The transgenic fruit ripened ~5 days earlier than the wild type (Fig. 2b), suggesting that SlGRAS4 acts as a novel regulator of tomato fruit ripening. In this study, a much higher carotenoid content was observed in *SlGRAS4*-OE fruit than in WT fruit at 40 DPA (Fig. 2c), which is consistent with the earlier ripening phenotype. Accordingly, we found that the expression level of *SlPSY1* in *SlGRAS4*-OE fruit was much higher than that in WT (Fig. 2d), which was also consistent with the increased carotenoid content phenotype in OE fruit.

Early studies have demonstrated the importance of ethylene biosynthesis genes during fruit ripening; the antisense activity of tomato *ACS2* or downregulation of *ACO1* in tomato plants causes reduced ethylene biosynthesis, and fruit ripening is thus retarded^37–39^. Similar to the result for *SlMADS1*, suppression of *SlMBP8* accelerated fruit ripening, and more ethylene production and enhanced *SlACS2*, *SlACO1*, and *SlACO3* expression were found in *SlMBP8*-silenced fruit^22^. On the other hand, delayed ripening was also observed in *SlCMB1*-downregulated tomato fruit, *SlACS2*, *SlACS4*, *SlACO1*, and *SlACO3* were repressed, and ethylene production and carotenoid content were all reduced^40^. Downregulation of *SlNAC48* and *SlNAC19* inhibits fruit ripening, and *SlACS2*, *SlACS4*, and *SlACO1* are seriously repressed^41^. These studies indicated that the effect of ripening regulators from different transcription factor families on fruit ripening may occur through the regulation of ethylene biosynthesis genes. Similarly, in our study, the expression levels of *SlACS2*, *SlACS4*, *SlACO1*, and *SlACO3* were all enhanced in *SlGRAS4*-OE fruit, and no changes in other ethylene biosynthesis genes were observed in OE fruit (Fig. 3, S2), suggesting that the earlier ripening of *SlGRAS4*-OE fruit may be caused by the higher level of these four crucial ethylene biosynthesis genes. In addition, the color turning of *SlGRAS4*-OE fruit after ethylene treatment occurred earlier than that of WT fruit, which was consistent with the increased expression levels of *SlACS2*, *SlACS4*, *SlACO1*, and *SlACO3* (Fig. 4). Furthermore, the ripening of WT fruit was severely inhibited by 1-MCP, whereas *SlGRAS4*-OE fruit ripened naturally after 1-MCP treatment, more endogenous ethylene was detected in *SlGRAS4*-OE fruit after 1-MCP treatment (Fig. S5), and the expression levels of *SlACS2*, *SlACS4*, *SlACO1*, and *SlACO3* were significantly higher than those in WT fruit after 1-MCP treatment (Fig. 4). These results indicated that overexpression of *SlGRAS4* accelerates fruit ripening by enhancing the expression of *SlACS2*, *SlACS4*, *SlACO1*, and *SlACO3*. In addition, *SlGRAS4*-OE seedlings exhibited more intense phenotypes after exogenous ACC treatment, and the expression of *SlACS2*, *SlACS4*, *SlACO1*, and *SlACO3* in OE seedlings was much higher than that in WT seedlings after ACC treatment (Fig. 5). These results will help us determine whether there is an interaction between SlGRAS4 and these ethylene biosynthesis genes.

Recently, many transcription factors involved in the regulation of fruit ripening through the regulation of ethylene biosynthesis genes in a direct manner have been reported. For example, MaERF11 can suppress the expression of *MaACS1* and *MaACO1* by directly binding to their promoters^42^. The banana C2H2 zinc-finger protein 1/2 can bind to the *MaACO1* promoter and repress its expression^43^. In kiwifruit, AdEIL2 and AdEIL3 can activate *AdACO1* expression to affect ripening^44^. In tomato, RIN can interact with the promoters of *SlACS2* and *SlACS4*^9–12^. FUL1 and FUL2 can bind to the *SlACS2*, *SlACS4*, and *RIN* promoters^20^. TAGL1 can directly bind to the promoter region of *SlACS2*, thus regulating ethylene biosynthesis^19,45^. LeHB-1 can directly regulate *SlACO1*, thus influencing fruit ripening^46^. Moreover, NOR-like1 influences ethylene biosynthesis in tomato fruit by regulating *SlACS2* and *SlACS4* in a direct manner^47^. However, no new direct regulator of the *ACS* and *ACO* genes has been identified in tomato. Our study showed that SlGRAS4 directly binds to and activates *SlACO1* and *SlACO3* promoters by yeast one-hybrid and dual-luciferase assays (Fig. 6), identifying a novel regulator of fruit ripening that directly modulates the expression of ethylene biosynthesis genes.

On the other hand, MADS-box transcription factors are key regulators of tomato fruit ripening and are usually formed as complexes by protein-protein interactions. Several MADS-box transcription factors, including SlMADS1, SlCMB1, and SlMBP8, can interact with RIN^21,22,40^. SlNAC4 can also interact with RIN^24^. However, there was no protein-protein interaction between SlGRAS4 and RIN, and considering that SlGRAS4 cannot interact with the *SlACS2* and *SlACS4* promoters, the increased expression of *SlACS2* and *SlACS4* in *SlGRAS4*-OE fruit may be caused by the enhanced expression level of *RIN* (Fig. 3f). Furthermore, there are three RIN-binding motifs in the *SlGRAS4* promoter (Table S2), but a yeast one-hybrid assay confirmed that there was no interaction between RIN and the *SlGRAS4* promoter region, suggesting that there was no direct regulatory relationship between RIN and *SlGRAS4*. In addition, the downregulated expression of *SlMADS1* that was directly repressed by SlGRAS4 (Figs. 3h, 7) also contributed to the enhanced *ACS* and *ACO* gene expression in OE fruit.

Notably, there were no obvious changes in the fruit ripening process of *SlGRAS4*-RNAi fruit compared to that of WT fruit. We obtained three effective *SlGRAS4-*repressing lines (Fig. S7), and four *SlGRAS4* homologous genes had no influence on *SlGRAS4-*RNAi fruit (Fig. S8). There were no differences in the days from anthesis to breaker stage between WT and *SlGRAS4*-RNAi plants (Fig. S9a). The SlGRAS4 target genes *SlACO1* and *SlACO3* were both repressed in RNAi fruit at the Br+3 stage, and the expression of *SlACS2* and *SlACO3* in RNAi fruit was significantly lower than that in WT fruit at the breaker stage. RIN was also downregulated in RNAi fruit at the Br + 3 stage, and the expression of *SlMADS1* in RNAi fruit showed an increasing trend (Fig. S9). The expression level of *SlACS4* was significantly enhanced in *SlGRAS4*-RNAi fruit at the Br + 3 stage (Fig. S9), and the expression of other ethylene biosynthesis genes, including *SlACS1a*, *SlACS3*, *SlACS6*, *SlACO2*, and *SlACO4*, was significantly increased in RNAi fruit at the breaker stage (Fig. S10). Similar to OE fruit, the expression levels of ethylene signaling genes also showed no changes in RNAi fruit compared to WT fruit (Fig. S11), except that *SlEBF3* was upregulated, but this gene was also upregulated in OE fruit. The expression levels of ripening-related transcription factors, including *SlPG2a*, *SlFUL1*, *SlFUL2*, *SlTAGL1*, *SlHB1*, and *CNR*, also showed no changes in *SlGRAS4*-RNAi fruit compared to WT fruit (Fig. S12). These results for *SlGRAS4*-RNAi fruit indicated that although downregulation of *SlGRAS4* resulted in the repression of *SlACO1* and *SlACO3*, the expression levels of other ethylene biosynthesis genes were significantly upregulated, leading to normal ripening of *SlGRAS4*-RNAi fruit, which may be caused by other fruit ripening regulators, as discussed above, that function in a complementary manner.

In our previous study, SlGRAS4 was identified as a positive regulator of fruit chilling tolerance. After chilling treatment, WT fruit could not turn red completely, whereas *SlGRAS4*-OE fruit ripened normally^31^. In addition to the metabolic pathways related to chilling tolerance regulated by SlGRAS4, as presented in our previous study, we hold the opinion that ethylene biosynthesis regulated by SlGRAS4 also contributes to the normal ripening of *SlGRAS4*-OE fruit after chilling treatment. Whether SlGRAS4 participates in the convergence of the fruit ripening process and cold response needs to be further studied, as they are both very complex regulatory networks. Overall, our work revealed a novel regulator (SlGRAS4) of fruit ripening and provided new insight into the complex network associated with fruit ripening.

## Materials and methods

### Plant materials and growth conditions

Tomato plants (*Solanum lycopersicum* cv. Micro-Tom) and transgenic lines in this background (three OE lines OE #12, OE #18, and OE #27 and three RNAi lines RNAi #10, RNAi #15 and RNAi #16 were used as in our previous study^31^) were grown in a greenhouse under controlled conditions (18-h light/6-h dark cycles, 25 °C day/18 °C night, and 60% relative humidity). For tissue expression analysis, 8 DPA fruit, 16 DPA fruit, MG, Br, 3 days post breaker (Br + 3) fruit, and 7 days post breaker (Br + 7) fruit were collected from six plants. Each tissue was sampled three independent times.

### Fruit ripening time and carotenoid determination

The ripening period was indicated as DPA, twelve plants of each line were included, and three independent observations were performed. The total carotenoid content was detected according to the method described previously with minor modification^48^. In brief, tomato fruit powder was extracted with acetone/hexane solution (2:3 by volume), and the supernatant was used for absorbance measurements at 663, 645, and 470 nm. The total carotenoid content was calculated by the following equation: total carotenoid (μg/g) = [1000 × A470 − 3.27 × (12.72 × A663 − 2.59 × A645) − 104 × (22.88 × A645 − 4.67 × A663)] × 10/229.

### Ethylene production measurement

The measurement of fruit ethylene production was performed according to a previous study^49^. In brief, fruit was placed in 50-mL airtight containers for 16 h, and then 1 mL of gas was injected into a gas chromatograph (Varian CP-3800 GC gas chromatograph, USA). Ethylene production was normalized to fruit weight, and ethylene standard gas was used as a control. The measurement was carried out with at least 10 fruit for each line, and three independent biological replicates were performed.

### Ethylene and 1-MCP treatment of tomato fruit

For *SlGRAS4* ethylene response testing, WT MG fruit was dipped in 10 ppm ethephon solution for 6 h, and WT Br was harvested and treated with 1-MCP (1 mg/L) for 1 h. For phenotypic observation, WT and transgenic MG fruit were dipped in 10 ppm ethephon solution for 3 h once a day for three days. Fruit at the breaker stage were harvested and treated with 1-MCP (1 mg/L) for three days. The fruit was placed in an incubator under a 18-h light (25 °C)/6-h dark (18 °C) cycle. The differences in color were observed, and the fruit were frozen in liquid nitrogen and stored at −80 °C after 72 h of treatment.

### Triple response assay

WT and transgenic seeds were sterilized and sown on ½ × MS alone and ½ × MS containing 2.0 μM ACC and then incubated in the dark for 7 days. The hypocotyl length and root length were assessed for the seedling triple response. For each line, at least 20 seedlings were measured, and three independent treatments were performed.

### Expression analyses by qRT-PCR

Total RNA extraction, first-strand cDNA synthesis and quantitative real-time PCR were performed using commercial kits following the manufacturer’s instructions (TAKARA, Japan). The PCR procedure was performed using a Bio-Rad CFX system (Bio-Rad, USA). The 2^−ΔΔCt^ method was used for calculation of the relative fold change by Bio-Rad CFX Manager 3.0, and *SlActin* was used as an internal reference gene. Three biological replicates were performed for each sample. All the primers used for qRT-PCR are listed in Table S3.

### Yeast one-hybrid assay

The yeast one-hybrid assay was performed according to our previous study^31^. In brief, *SlACO1*, *SlACO3*, and *SlMADS1* promoter regions containing the SlGRAS4-binding motif were cloned into the pAbAi vector and transformed into the Y1HGold yeast strain. After screening the inhibitory aureobasidin A concentration, the SlGRAS4-pGADT7 plasmid and empty pGADT7 plasmid were transformed into the recombinant yeast strain. The interaction between SlGRAS4 and promoter regions was determined according to the growth of the colony on SD/-Leu/AbA culture medium.

### Dual-luciferase assay

The full-length SlGRAS4 ORF was cloned into the pGreenII 62-SK vector, which was transformed into *Agrobacterium tumefaciens* strain GV3101 as an effector. The promoter of the target genes was cloned into the pGreenII 0800-LUC vector, which was transformed into GV3101 as a reporter. The effector and reporter strains were cotransfected into tobacco (*Nicotiana benthamiana*) leaves, and the commercial Dual-Luciferase^®^ Reporter Assay System (Promega, USA) was used for LUC assays.

## Supplementary information

Supplementary information

## Data Availability

All relevant data are provided within the paper and its supplementary files.
