# Supplementary material for: SlGRAS4 accelerates fruit ripening by regulating ethylene biosynthesis genes and SlMADS1 in tomato
Source: Hortic Res. 2021 Jan 1;8:3. doi: 10.1038/s41438-020-00431-9 (PMC7775462; doi:10.1038/s41438-020-00431-9)
Supplement: Supplementary file 1 — Supplementary information [file 41438_2020_431_MOESM1_ESM.doc]

**Table S1 Putative SlGRAS4-binding motif present in the promoter regions of *SlACO*, *SlACS* genes and *SlMADS1*.**

| **Gene** | **Position** | **Sequence** |
| --- | --- | --- |
| ***SlACO1*** | -996 | AGGCATGT |
|  | -910 | TCTCATGT |
|  | -857 | TCTCATGT |
|  | -802 | AACCACGT |
|  | -753 | ACTCGTGG |
|  | -721 | TTCCGCGT |
|  | -711 | ACACCTGC |
|  | -649 | ACTCGTGA |
|  | -379 | ACACATGA |
|  | -339 | AGCCATGG |
|  | -322 | CATCATGT |
| ***SlACO3*** | -1628 | CATCGCGT |
|  | -1558 | CAACCGGT |
|  | -1528 | CGTCCGGA |
|  | -1518 | CATCGCGT |
|  | -1501 | TATCCGGC |
|  | -1466 | CGTCCCGA |
|  | -1456 | CATCGCGT |
|  | -1394 | CGTCCTGA |
|  | -1380 | CATCATGT |
|  | -1360 | ATTCATGA |
|  | -1108 | TATCATGT |
|  | -1014 | CTACACGC |
|  | -957 | AGACATGG |
|  | -816 | AATCATGC |
|  | -699 | TATCGTGC |
|  | -675 | TCATGCAC |
|  | -563 | AGTCACGT |
|  | -408 | ATACAGGT |
| ***SlMADS1*** | -1618 | TTGCATGT |
|  | -1580 | TATCATGT |
|  | -763 | CAACGCGT |
|  | -617 | GTACGTGC |
|  | -405 | TGCCCTGC |
|  | -285 | TGTCACGT |
|  | -83 | TGACATGA |
|  | -75 | TCGCACGT |
|  | -64 | TAACGTGA |
| ***SlACS2*** | -1500 | AATCATGA |
|  | -1472 | ACACCTGC |
|  | -1394 | GCCGGCTA |
|  | -1268 | TCCCAGGA |
|  | -1148 | TCTCACGT |
|  | -1126 | GAACATGA |
|  | -903 | TTGCAGGA |
|  | -760 | TGACAGGG |
|  | -239 | CGACATGT |
| ***SlACS4*** | -919 | AATCGTGA |
|  | -680 | AGACGTGG |
|  | -349 | CGACGGGT |
|  | -292 | TGCCATGT |
|  | -262 | TTTCATGC |
| For each gene, the 1.8 Kb genomic sequence upstream of the translation start codon (ATG) was analyzed. | | |

***Table S2 Putative RIN-binding motif present in the promoter region of SlGRAS4.***

| **Gene** | **Position** | **Sequence** |
| --- | --- | --- |
| ***SlGRAS4*** | -862 | CAAATATTTG |
|  | -818 | CTTTTTAAAG |
|  | -117 | CTATTTTTTG |
| RIN-binding motif CArG box [C(A/T)8G] was searched in the *SlGRAS4* promoter. The 1.5 Kb genomic sequence upstream of the translation start codon (ATG) was analyzed. | | |

**Table S3 Primers used in this study.**

| **Primers used for DNA constructs** | | |
| --- | --- | --- |
| Primer Name | Forward (5’-3’) | Reverse (5’-3’) |
| SlGRAS4-AD | GAATTCATGGAAGCCCTTTTTCAAG | GGATCCGACTAATTTTACAGACTGCTT |
| SlGRAS4-62SK | GGATCCATGGAAGCCCTTTTTCAAG | AAGCTTCTAGACTAATTTTACAGACTGCTT |
| SlACO1-pAbAi | GGTACCGGTCGATTGACCGAACCACGT | GTCGACTAAGCAGGTGTAAACGCGGA |
| SlACO1-0800 | GGTACCGGTTTAGGATTCTATAAATAGAGGC | AAGCTTCTTAATTTCTTGGTAAAGTGTTTTC |
| SlACO3-pAbAi | GGTACCGTATTTGTTATCTCCTATCA | GTCGACTAGTGCAGAACTTCCATGTC |
| SlACO3-0800 | GGTACCGAATATATTCCAAAACATTTAGATA | AAGCTTCTTAATTTTTCTTGGTAATTAAATT |
| SlMADS1-pAbAi | GGTACCTGATTGTGTCACGTGTCTCT | GTCGACTAATATCACGTTAGAAACGT |
| SlMADS1-0800 | GGTACCAAGCCTATTATAATATGAGTTAAAT | AAGCTTAACAAAGAGCATCTAACTATTAATA |
| **Primers used for q-RT-PCR** | | |
| Gene Name | Forward (5’-3’) | Reverse (5’-3’) |
| *SlACS1a* | TCGTTTCGAAGATTGGATGA | CAACAACAACAAATCTAAGCCATT |
| *SlACS2* | TGTTAGCGTATGTATTGACAACTGG | TCATAACATAACTTCACTTTTGCATTC |
| *SlACS3* | CCCTTGTCCACAAATCCAGA | ACAGAGTGCACCCTCTAACATTT |
| *SlACS4* | CTCCTCAAATGGGGAGTACG | TTTTGTTTGCTCGCACTACG |
| *SlACS6* | CTCCTATGGTCCAAGCAAGG | CGACATGTCCATAATTGAACG |
| *SlACO1* | GCCAAAGAGCCAAGATTTGA | TTTTTAATTGAATTGGGATCTAAGC |
| *SlACO2* | TTTATTACAAAGTGTGCGTCCCTA | CTCATTTTTGGGTATTAAAATATGTGT |
| *SlACO3* | CTCCCATGCGCCACTCTATT | AGATCACCGCGTCATTTCCT |
| *SlACO4* | GGAGCCTAGGTTTGAAGCAA | AAACAAATTCCCCCTTGAAAA |
| *SlSAM1* | CGAGTCTGTGAACGAGGGTC | TGCAACTTTGCTCTCGGGAT |
| *SlETR1* | GGAAGAACATTGGCATTGGAAG | CCAACTGGATTTTGGTGTCGT |
| *SlETR2* | TTGGAGGAATCAATGAGGGC | TCATTACGCGCACGAACAG |
| *SlNR* | TGCTGTTCGTGTACCGCTTT | TCATCGGGAGAACCAGAACC |
| *SlETR4* | TGGAGGAGTGAGTGTGGATGC | ATGGCTGTCGTTCTTGGGC |
| *SlETR5* | GTGCTCTGGGCCCTTCACTA | GAACTTACGCACCCTCAATGC |
| *SlETR6* | TCAAAAAGCCGGTGATCTCG | GCACCCATTTGAACGGAAAA |
| *SlCTR1* | CGATTTGAACATGACAGGGAG | AAGGGATTGAGATGGAAGATGG |
| *SlEIN2* | GTGTGCTGAATAAGTTTAGTGGAG | TGCTGTACAATAGAAGAATGGAGG |
| *SlEIL2* | TGAAGATGATGGAAGTCTGTAAGG | CCACTCCCTGAGATTATCCGA |
| *SlEIL3* | ACAGGACTTCAAGAAACAACCA | GTGTTGTGCTCATAGTTGATCTG |
| *SlEBF1* | CTTAAACATCCAGCACTGCAAT | AACTTGTAAGTTGCATTGACCC |
| *SlEBF2* | CTGTCGTTCAATCTCCAACAAG | TTGGTGCAGTATTTTCCAACAG |
| *SlEBF3* | TTATTTTGGCTCTTGGTCATGC | GCAGCTGAAAGATTAGTATGGC |
| *RIN* | CAGCTTGAACGTCAATTGGAT | CTTTGCTCACCACAATGCCA |
| *NOR* | AGAGAACGATGCATGGAGGTTTGT | ACTGGCTCAGGAAATTGGCAATGG |
| *SlMADS1* | TTGCAGCACTTCAAGCATGG | ACGTGCCCAAATCTTCACCA |
| *SlPG2a* | AACGGACCACAATCTTGAC | CTGCTCGGAGTCTGAACC |
| *SlFUL1* | GTTTTGCCACAACAACTGGACTC | CTTGCTGCTGTGAAGAACTACC |
| *SlFUL2* | AATGGAGAAGTAGAAGGATCATCG | GATAACATAATATTGTCCGCTTGC |
| *SlTAGL1* | ACTTTCTGTTCTTTGTGATGCT | TTGGATGCTTCTTGCTGGTAG |
| *SlHB1* | CAATCGGAGGAAGATGATGG | TGTTCATGGTGCTGCTCTTC |
| *CNR* | ACATCCTTCTTGCCAGGTCG | GCCTTCGGCAACTCCTCTTA |
| *SlGRAS4* | TGTGTTACAAAGTGGCAGCA | GTCTGCAGCAACATCTCCTC |
| *SlGRAS30* | CTGTTGCTGTAGGTAATCAACG | GATGGTACGCTTTCAAGACATC |
| *SlGRAS36* | GTCGTGGTGAATTGTTTGTACA | ATGCATTGTACATAGCGTTGAC |
| *SlGRAS42* | TGAATCAACTCTACGATCCGAG | TGCGCACAATTTATCATGAGAG |
| *SlGRAS46* | ATGAGACCGTGTTATCTGACAG | AAAAATGAAACAGGACCTCACG |
| *SlActin* | TGTCCCTATTTACGAGGGTTATGC | CAGTTAAATCACGACCAGCAAGAT |


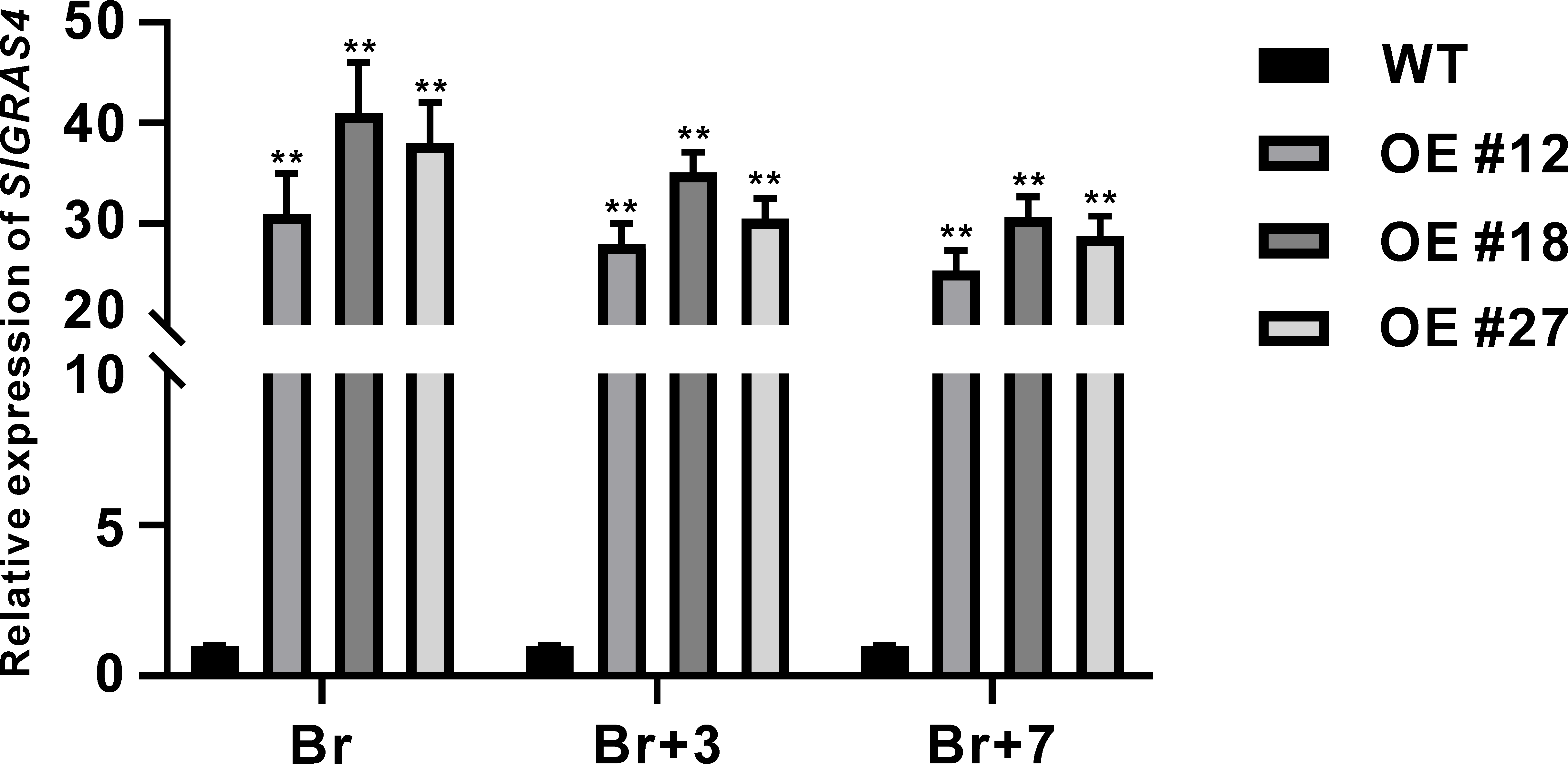


**Fig. S1** **The relative expression level of *SlGRAS4* in WT and overexpression fruit.**

The relative expression level of *SlGRAS4* in WT and overexpression fruit at Br, Br+3 and Br+7 stages, respectively. The relative expression level in WT fruit was set as 1 at different stages. Data are the mean values of three independent replicates and error bars show the s.d. Asterisks indicate significant differences between WT and transgenic lines (Two-tailed Student's *t*-test, ***P* < 0.01).


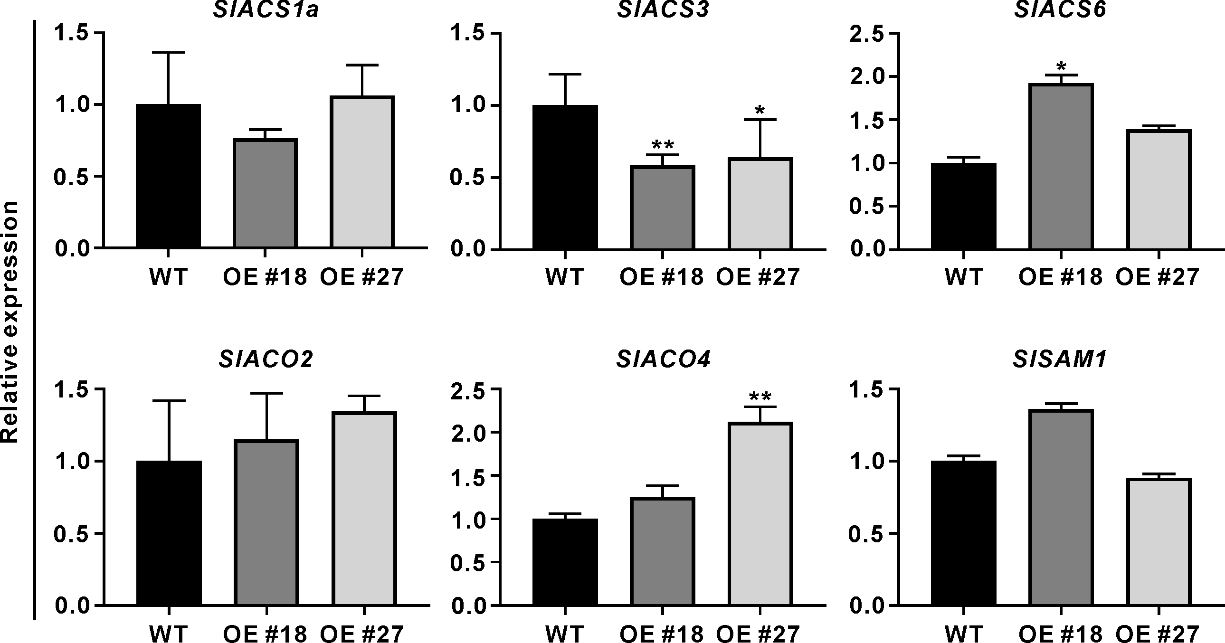


**Fig. S2** **The relative expression levels of other ethylene biosynthesis genes in WT and *SlGRAS4*-overexpressing fruit.**

The relative expression level of *SlACS1a*, *SlACS3*, *SlACS6*, *SlACO2*, *SlACO4*, and *SlSAM1* in WT and *SlGRAS4*-OE fruit at breaker stage was analyzed by qRT-PCR. Data are the mean values of three independent replicates and error bars show the s.d. Asterisks indicate significant differences between WT and transgenic lines (Two-tailed Student's *t*-test, **P* < 0.05, ***P* < 0.01).


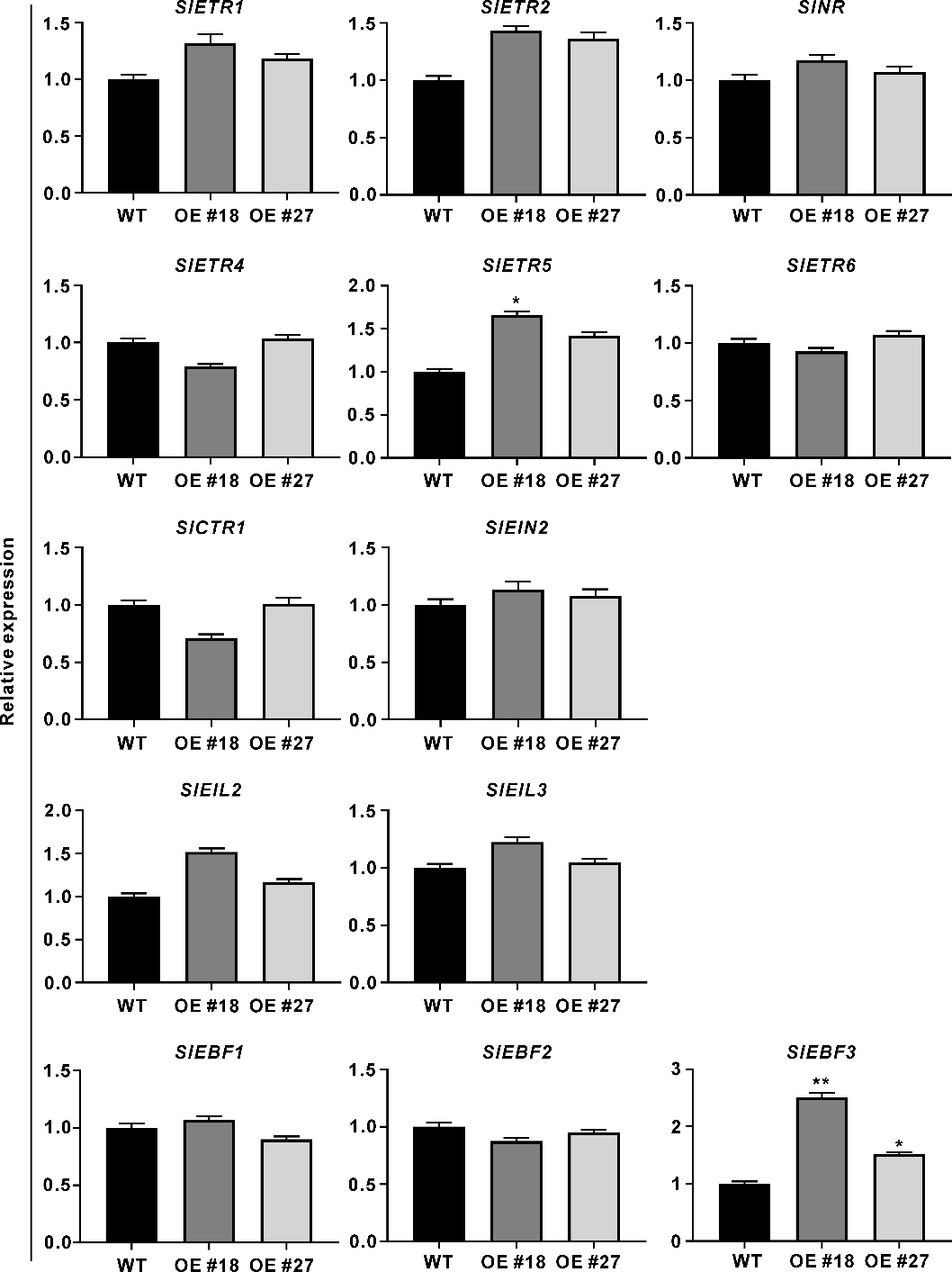


**Fig. S3 The relative expression levels of ethylene signaling genes in WT and *SlGRAS4*-overexpressing fruit.**

The relative expression level of *SlETR1*, *SlETR2*, *SlNR*, *SlETR4*, *SlETR5*, *SlETR6*, *SlCTR1*, *SlEIN2*, *SlEIL2*, *SlEIL3*, *SlEBF1*, *SlEBF2* and *SlEBF3* in WT and *SlGRAS4*-OE fruit at breaker stage was analyzed by qRT-PCR. Data are the mean values of three independent replicates and error bars show the s.d. Asterisks indicate significant differences between WT and transgenic lines (Two-tailed Student's *t*-test, **P* < 0.05, ***P* < 0.01).


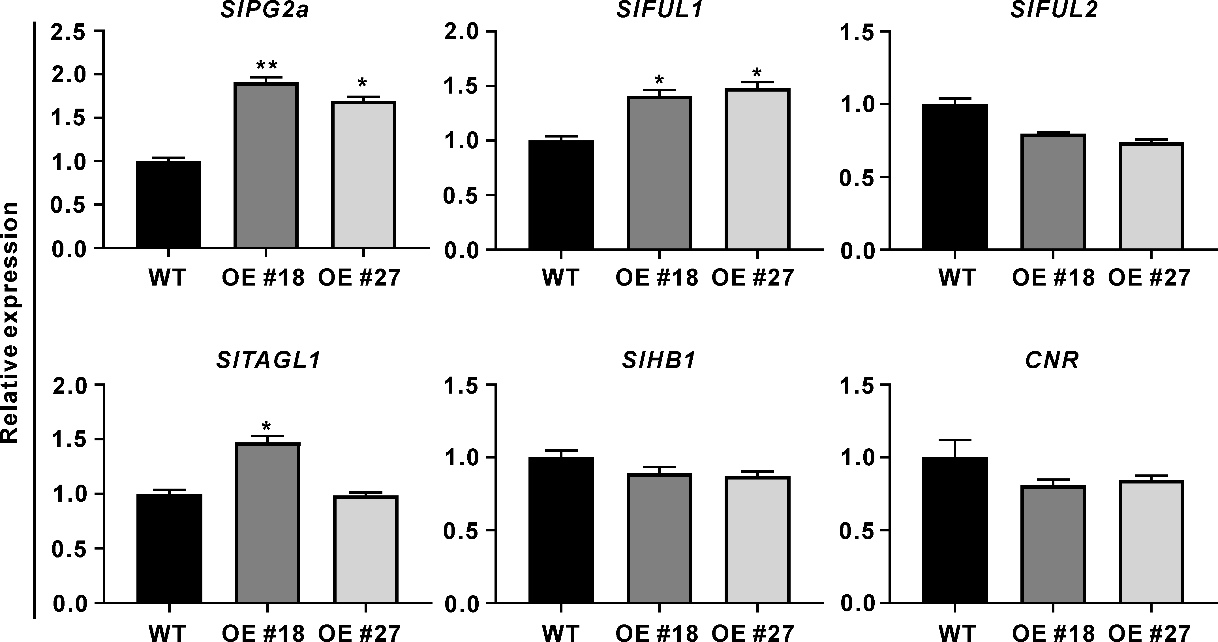


**Fig. S4** **The relative expression levels of ripening-related transcription factors in WT and *SlGRAS4*-overexpressing fruit.**

The relative expression level of *SlPG2a*, *SlFUL1*, *SlFUL2*, *SlTAGL1*, *SlHB1*, and *CNR* in WT and *SlGRAS4*-OE fruit at breaker stage was analyzed by qRT-PCR. Data are the mean values of three independent replicates and error bars show the s.d. Asterisks indicate significant differences between WT and transgenic lines (Two-tailed Student's *t*-test, **P* < 0.05, ***P* < 0.01).


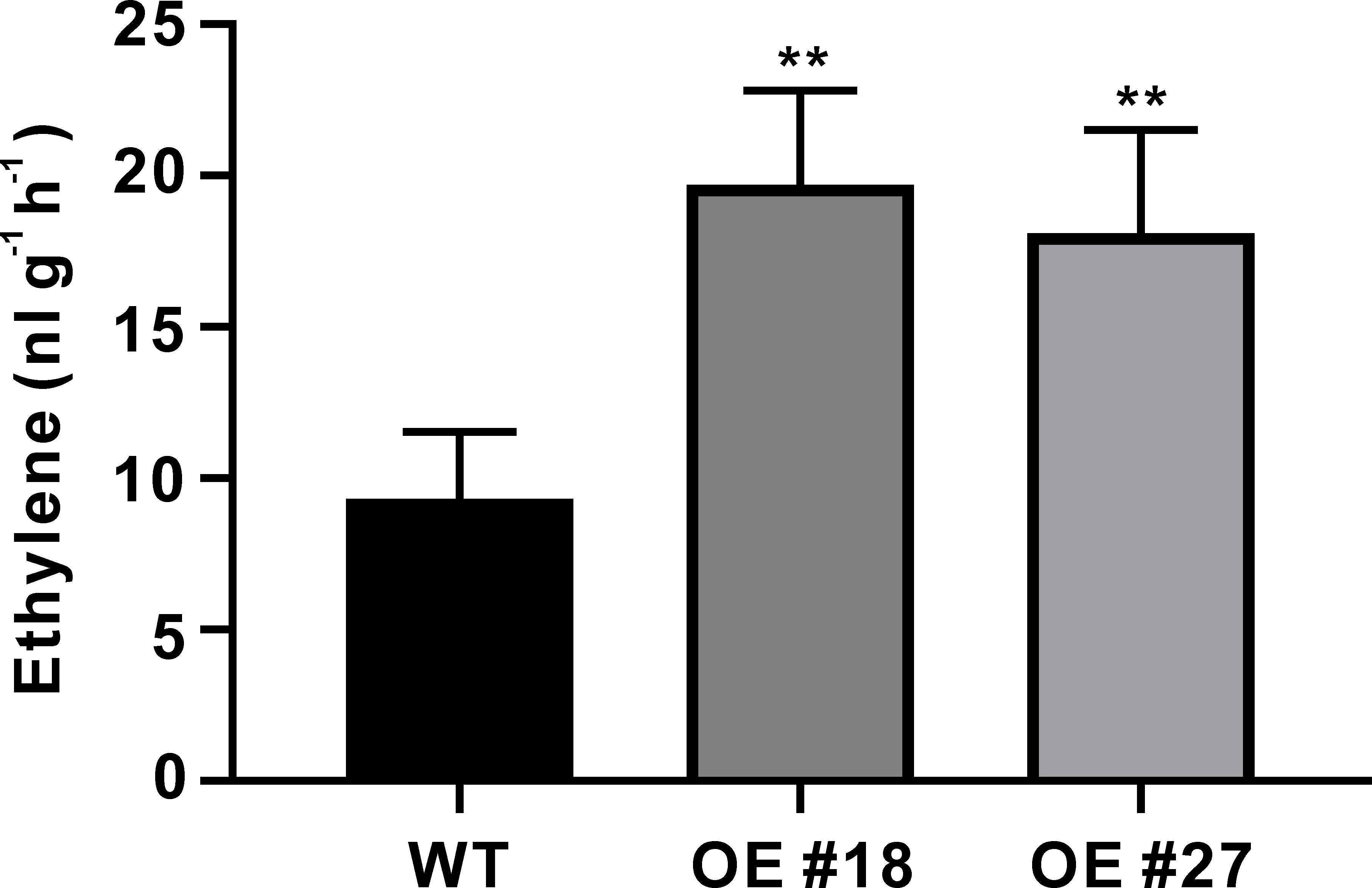


**Fig. S5 Ethylene measurement in WT and *SlGRAS4*-OE fruit after 1-MCP treatment.**

Data represent mean values of three independent experiments and error bars show the ± standard error. Asterisks indicate significant differences between WT and transgenic lines (Two-tailed Student's *t*-test, ***P* < 0.01).

**
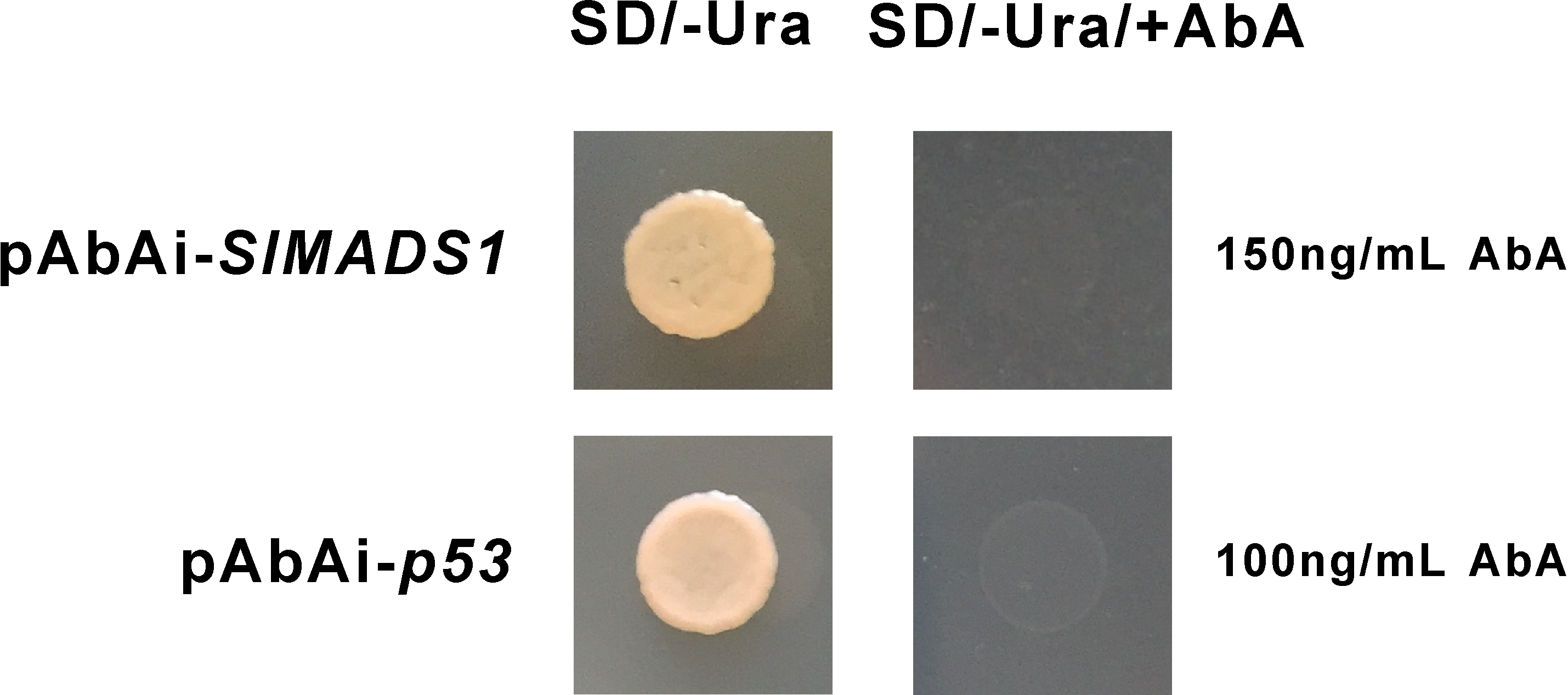
**

**Fig. S6 Inhibitory aureobasidin A concentration of the pAbAi-*SlMADS1* Y1HGold yeast strain.**

The pAbAi-*p53* Y1HGold yeast strain was control.

**
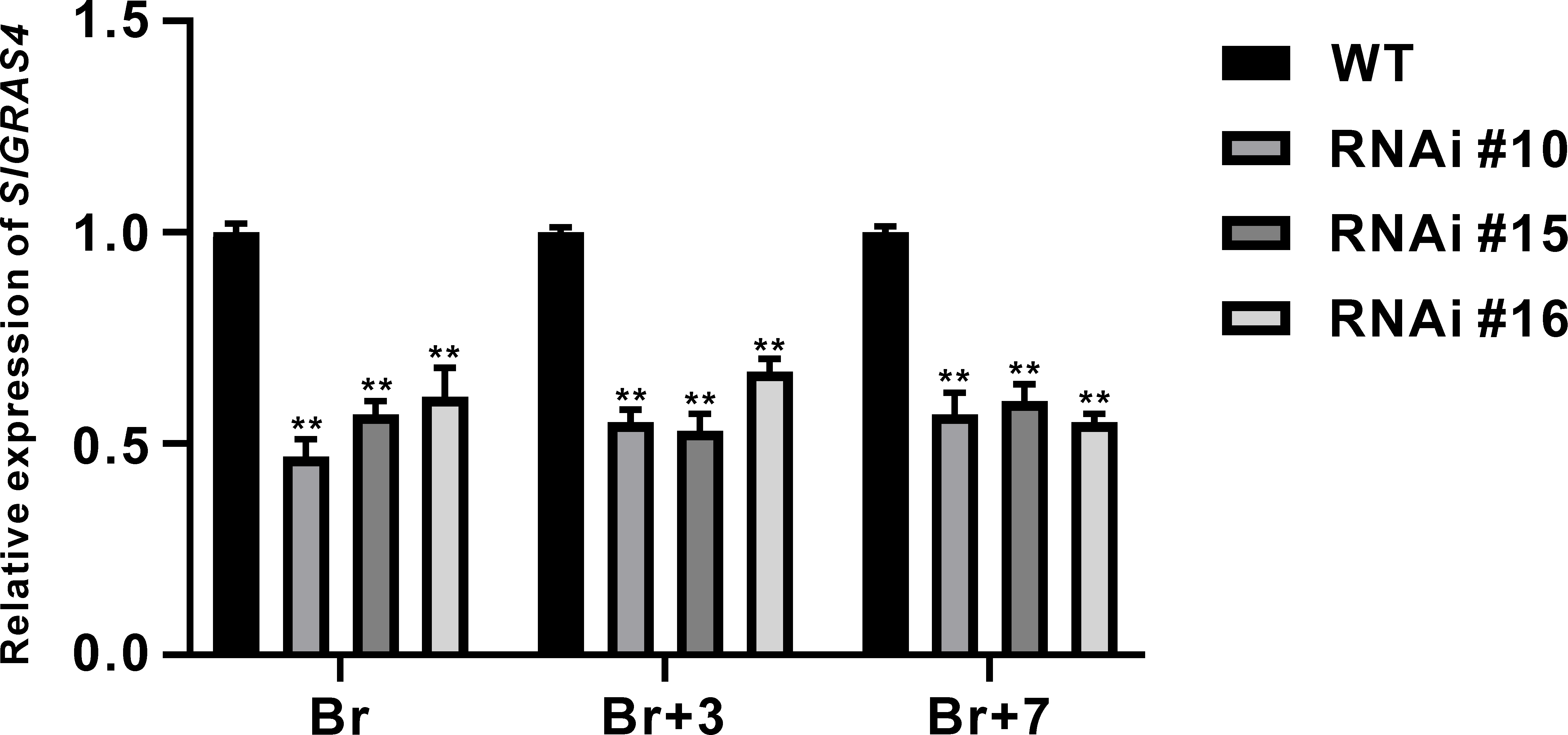
**

**Fig. S7 The relative expression level of *SlGRAS4* in WT and RNAi fruit.**

The relative expression level of *SlGRAS4* in WT and RNAi fruit at Br, Br+3 and Br+7 stages, respectively. The relative expression level in WT fruit was set as 1 at different stages. Data are the mean values of three independent replicates and error bars show the s.d. Asterisks indicate significant differences between WT and transgenic lines (Two-tailed Student's *t*-test, ***P* < 0.01).

**
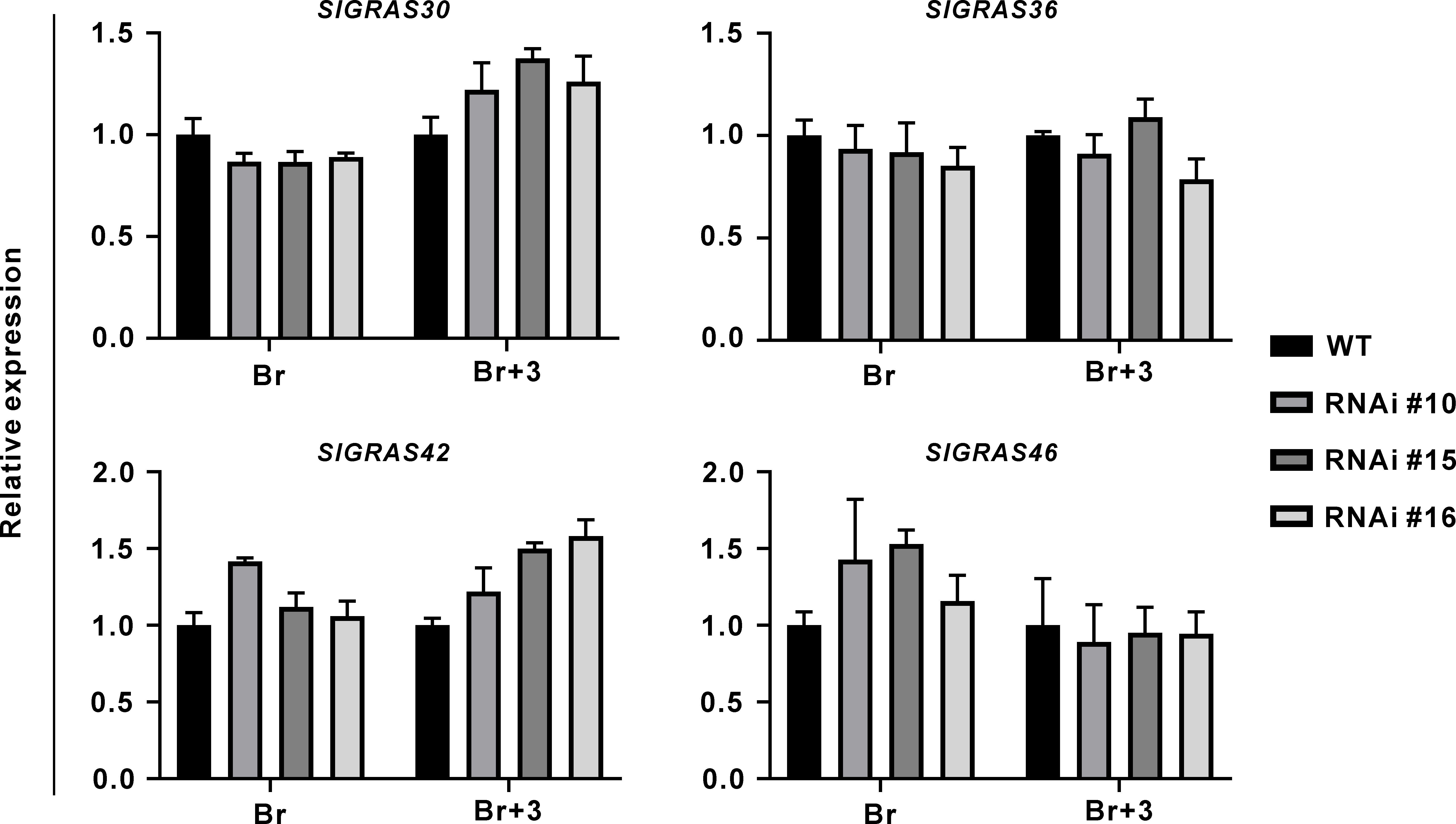
**

**Fig. S8 The relative expression levels of *SlGRAS4* homologous genes in WT and RNAi fruit.**

The relative expression level of *SlGRAS4* homologous genes *SlGRAS30*, *SlGRAS36*, *SlGRAS42* and *SlGRAS46* in WT and RNAi fruit at Br and Br+3 stages, respectively. The relative expression level in WT fruit was set as 1 at different stages. Data are the mean values of three independent replicates and error bars show the s.d.

**
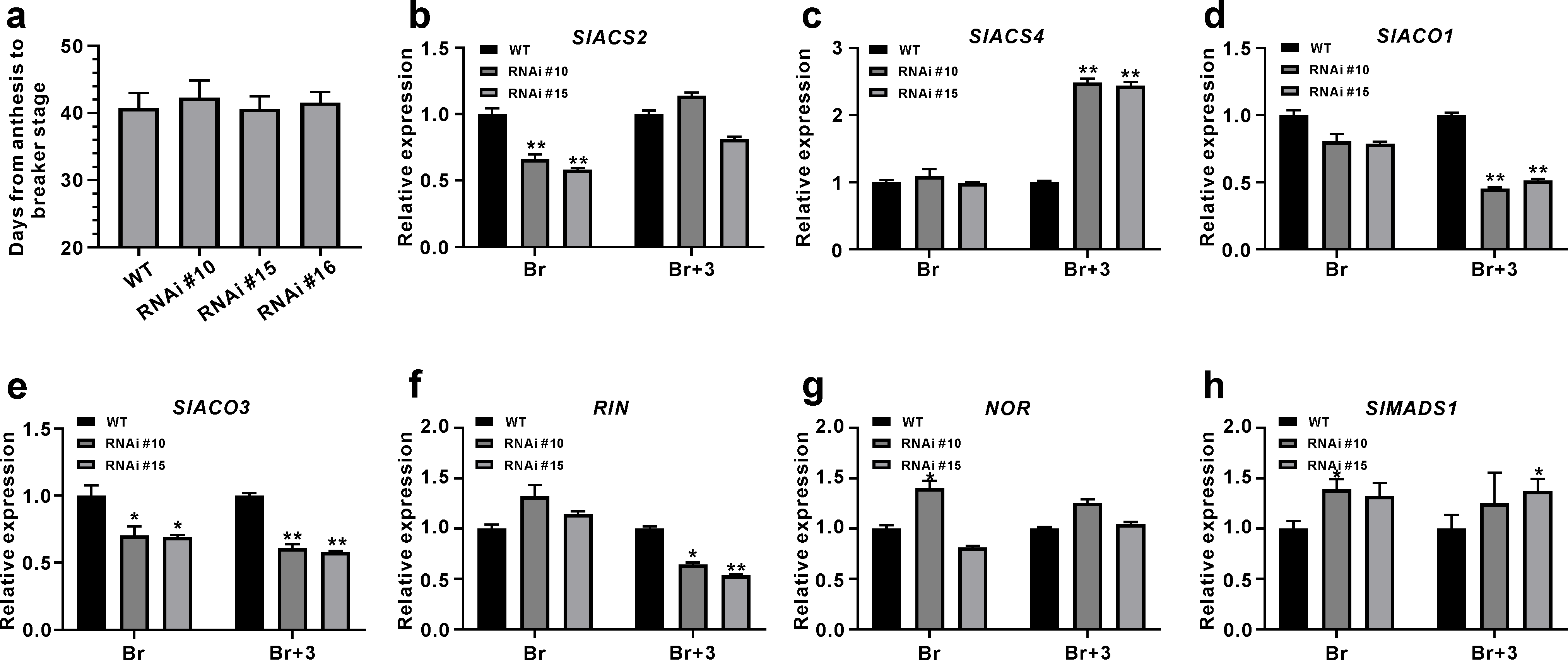
**

**Fig. S9 Ripening time of *SlGRAS4*-RNAi fruit and the expression of ethylene biosynthesis and ripening-related genes in WT and *SlGRAS4*-RNAi fruit.**

(a) Days from anthesis to breaker stage in WT and *SlGRAS4*-RNAi fruit. The expression level of *SlACS2* (b), *SlACS4* (c), *SlACO1* (d), *SlACO3* (e), *RIN* (f), *NOR* (g), and *SlMADS1* (h) was analyzed by qRT-PCR. Br, breaker stage; Br+3 (3 days post breaker). Data represent mean values of three independent experiments and error bars show the ± standard error. Asterisks indicate significant differences between WT and transgenic lines (Two-tailed Student's *t*-test, **P* < 0.05, ***P* < 0.01).

**
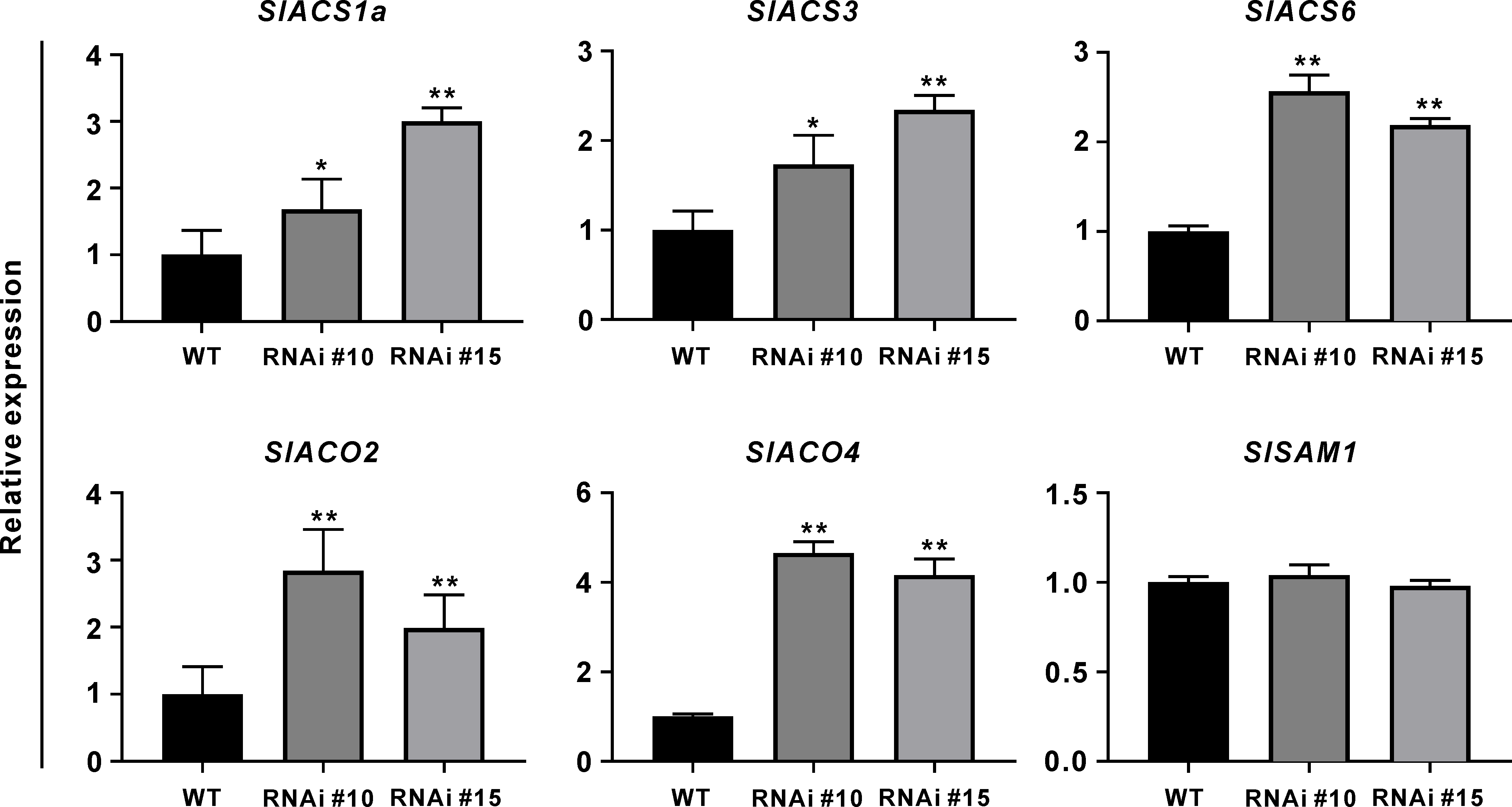
**

**Fig. S10 The relative expression levels of other ethylene biosynthesis genes in WT and *SlGRAS4*-RNAi fruit.**

The relative expression level of *SlACS1a*, *SlACS3*, *SlACS6*, *SlACO2*, *SlACO4*, and *SlSAM1* in WT and *SlGRAS4*-RNAi fruit at breaker stage was analyzed by qRT-PCR. Data are the mean values of three independent replicates and error bars show the s.d. Asterisks indicate significant differences between WT and transgenic lines (Two-tailed Student's *t*-test, **P* < 0.05, ***P* < 0.01).

**
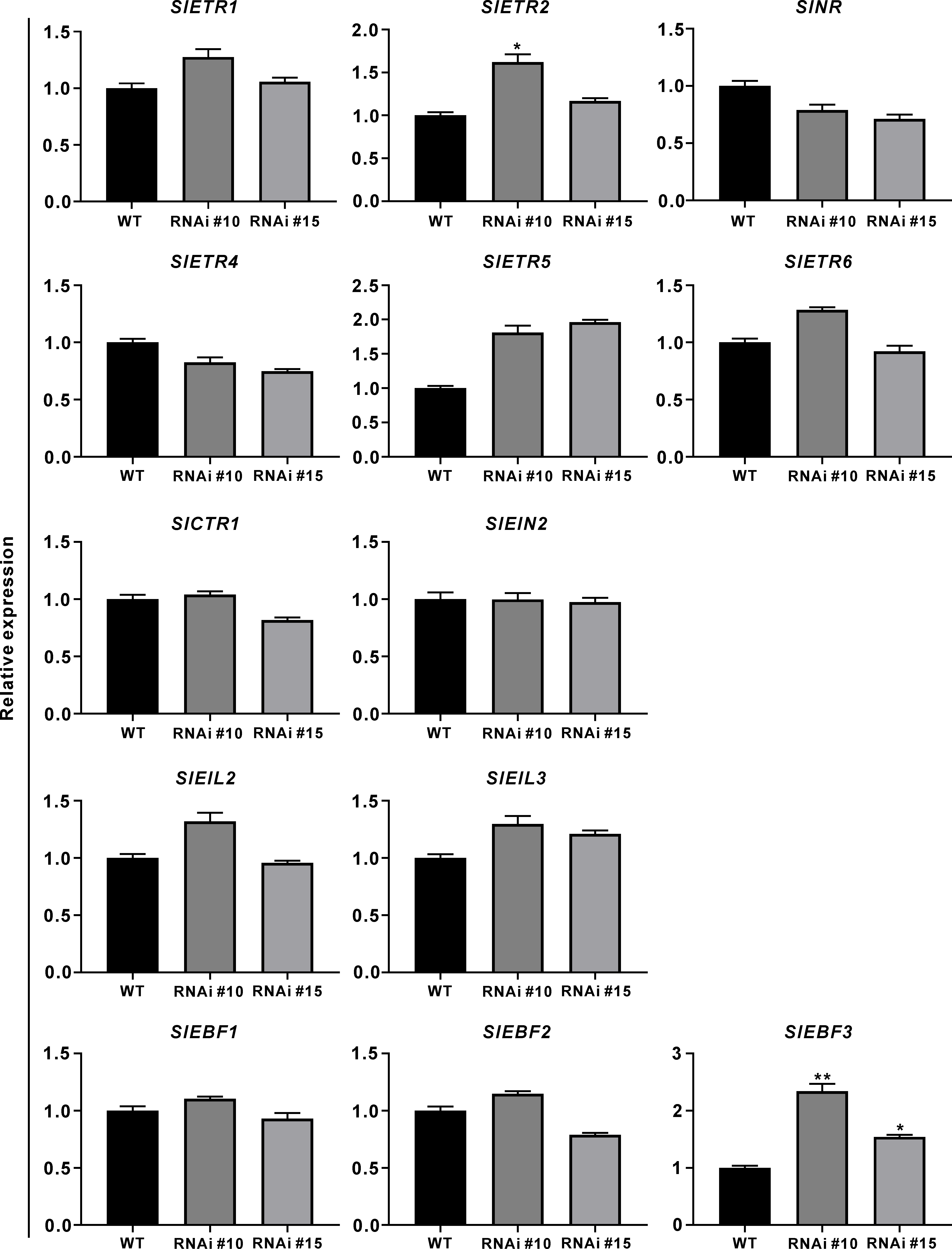
**

**Fig. S11 The relative expression levels of ethylene signaling genes in WT and *SlGRAS4*-RNAi fruit.**

The relative expression level of *SlETR1*, *SlETR2*, *SlNR*, *SlETR4*, *SlETR5*, *SlETR6*, *SlCTR1*, *SlEIN2*, *SlEIL2*, *SlEIL3*, *SlEBF1*, *SlEBF2* and *SlEBF3* in WT and *SlGRAS4*-RNAi fruit at breaker stage was analyzed by qRT-PCR. Data are the mean values of three independent replicates and error bars show the s.d. Asterisks indicate significant differences between WT and transgenic lines (Two-tailed Student's *t*-test, **P* < 0.05, ***P* < 0.01).

**
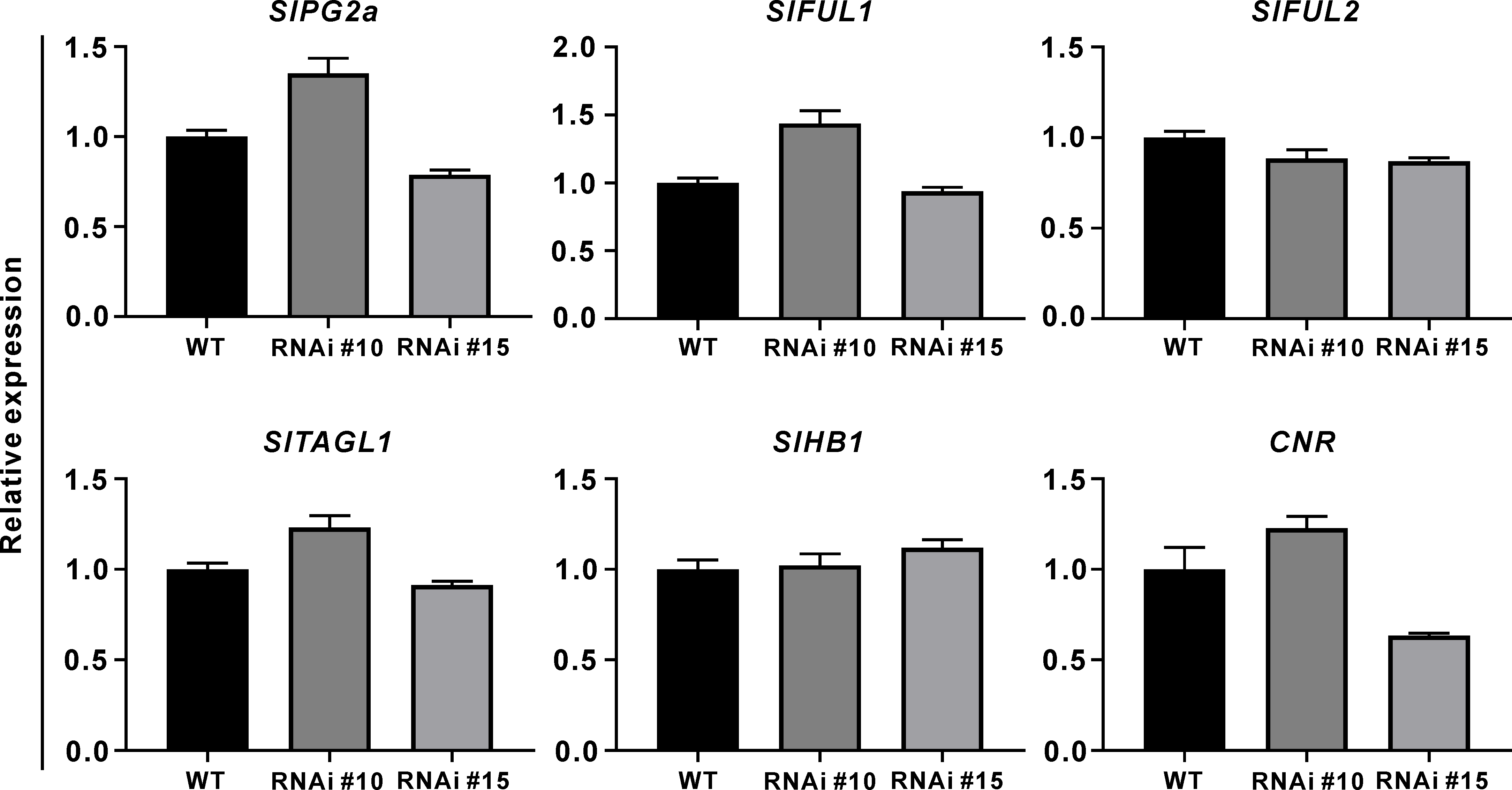
**

**Fig. S12 The relative expression levels of ripening-related transcription factors in WT and *SlGRAS4*-RNAi fruit.**

The relative expression level of *SlPG2a*, *SlFUL1*, *SlFUL2*, *SlTAGL1*, *SlHB1*, and *CNR* in WT and *SlGRAS4*-RNAi fruit at breaker stage was analyzed by qRT-PCR. Data are the mean values of three independent replicates and error bars show the s.d.
